# Combination of NF-kB targeted siRNA and methotrexate in a hybrid nanocarrier towards the effective treatment in rheumatoid arthritis

**DOI:** 10.1186/s12951-018-0382-x

**Published:** 2018-07-30

**Authors:** Weifeng Duan, Huan Li

**Affiliations:** 1 Department of Limb Function Rehabilitation, Luoyang Orthopedic Hospital of Henan Province, Luoyang, 471000 Henan Sheng China; 2 Nursing College, Henan University of Chinese Medicine, Zhengzhou, 450000 Henan Sheng China

**Keywords:** Rheumatoid arthritis, Methotrexate, siRNA, Liposome, Calcium phosphate, Inflammation

## Abstract

**Background:**

The transcription factor NF-kB plays an important role in the pathogenesis of rheumatoid arthritis (RA). Effective treatment of RA is hindered due to the lack of specificity of small molecules in the inflamed joints. In this study, we aimed to develop a unique hybrid-nanoparticles system comprised of calcium phosphate/liposome to deliver NF-kB-targeted siRNA and methotrexate (MTX) to diseased site.

**Results:**

We have successfully demonstrated that the combination of siRNA and MTX in a calcium phosphate/liposome-based hybrid nanocarrier could effectively treat the RA. We have showed that folate receptor-targeted nanocarrier system significantly suppression the arthritis progression in mice model. Substantial accumulation of F-siRML was observed in LPS-activated macrophages. These kind of activated macrophages are generally present in the RA and osteoarthritis and folate-targeted nanoparticle enables the effective accumulation of therapeutics in the diseased site. The combinational nanoparticles effectively blocked the NF-kB signaling pathways and reduced the expression of pro-inflammatory cytokines. Furthermore, siRML and F-siRML did not show any decrease in the lymphocyte count indicating that it can avoid the adverse effect of MTX.

**Conclusion:**

Therefore, siRML and F-siRML provides unique benefits of excellent therapeutic efficacy with excellent safety profile in the arthritic mice and could be an promising approach in the treatment of rheumatoid arthritis.

**Electronic supplementary material:**

The online version of this article (10.1186/s12951-018-0382-x) contains supplementary material, which is available to authorized users.

## Background

Rheumatoid arthritis (RA) is generally associated with high morbidity and mortality rate and affects nearly 1% of the global populations [1]. In RA, immune cells and activated macrophages infiltrate in the synovial tissue and destroy the cartilage and activate the inflammatory signaling pathways [2]. This process results in progressive destruction of bone and cartilage and cause immense pain and disability and there it is called autoimmune disorder [3]. The macrophage (M1) that entered the joints secretes large amount of inflammatory cytokines including TNF-α and IL-1β and results in the progression of RA [4, 5]. On the other wise, macrophage M2 acts as an anti-inflammatory factor and actively involved in tissue repair. Therefore, converting the M1 to M2 in the joints would be an attractive approach for the treatment of RA [6]. It has been reported that NH-kB signaling is actively involved in the polarization of macrophages and therefore inhibition of this signaling pathways could be effective way to increase the level of M2 in the joints [7].

It is well known that NF-kB locates in the cytoplasm and present as an inactive complex with kB proteins. In RA, the inflammatory signals from TNF-α and IL-1β degrades the inhibitory kB protein and thereby NF-kB gets liberated and transfer to the nucleus where they activate several inflammatory signals [8]. This process leads to the cartilage damage and further leads to severe RA progression [9]. Earlier, several small molecule inhibitor of NF-kB was developed but none result resulted satisfactory outcome. Off late, small-interfering RNA (siRNA) showed enormous potential to downregulate key cytokines such as TNF-α and IL-1β [10, 11]. In this study, we have selected p65 siRNA that directly targets the NF-kB family member p65 and could potentially inhibit the NF-kB-based inflammatory activities [13]. We expected that siRNA could potentially downregulate the p65 and thereby M1 macrophages re-polarize into M2 macrophages which are involved in tissue repair. As it is highly unlikely that siRNA alone could not block the complex inflammatory signal process, we have combined the siRNA with an antirheumatoid drug—methotrexate (MTX) [12, 14]. MTX has been used in the treatment of RA since decades either as a single agent or in combination [15]. However, long term use of MTX results in adverse effect and drug-resistance [16, 17]. Therefore, a combination of siRNA + MTX could elicit the synergistic effect in the treatment of RA.

The development of drug delivery system has been reported to enhance the performance of therapeutics [18]. The loading of therapeutics in the nanoparticle system is expected to promote the circulation in the blood circulation as well as promote the permeability in the biomembrane [19]. Several approaches have been developed for siRNA delivery such as polyethylenimine (PEI), dendrimers, or chitosan [20, 21]. Such complexes have been shown to protect the siRNA and mediate the cellular uptake in the cancer cells; however, strong positive charge is detrimental to the bioactivity of siRNA. Therefore, in this study, we have prepared the calcium phosphate nanoparticles (CaP) for the delivery of siRNA. The CaP is highly biocompatible and biodegradable and could help increase the performance of siRNA [22]. In order to further increase the stability of siRNA/CaP complex and to load the MTX, we have designed the PEGylated liposome that could load the siRNA/CaP in its core while MTX will be loaded in the lipid shell of the liposomes. The main advantage of liposome includes the high drug-to-carrier ratios, compatibility of lipids, high stability and physical entrapment of therapeutics without any need for complicated chemistry [23]. The chemical conjugations not only decrease the activity of therapeutics but also delay the release of conjugated molecules [24]. Furthermore, PEGylated liposome was conjugated with folic acid as a target ligand in order to target the folate receptorβ (FRβ) in the activated macrophages [25].

Here, we examined the potential of combination of NH-Kb-specific siRNA + MTX in the treatment of rheumatoid arthritis. For this purpose, siRNA/CaP was prepared and loaded along with MTX in a folate conjugated PEGylated liposome. The formulations were intravenously injected in the collagen-induced arthritic mice and therapeutic efficacy was evaluated in terms of arthritis score, paw thickness and inflammatory markers level in the serum.

## Results and discussion

**Fig. 1:**
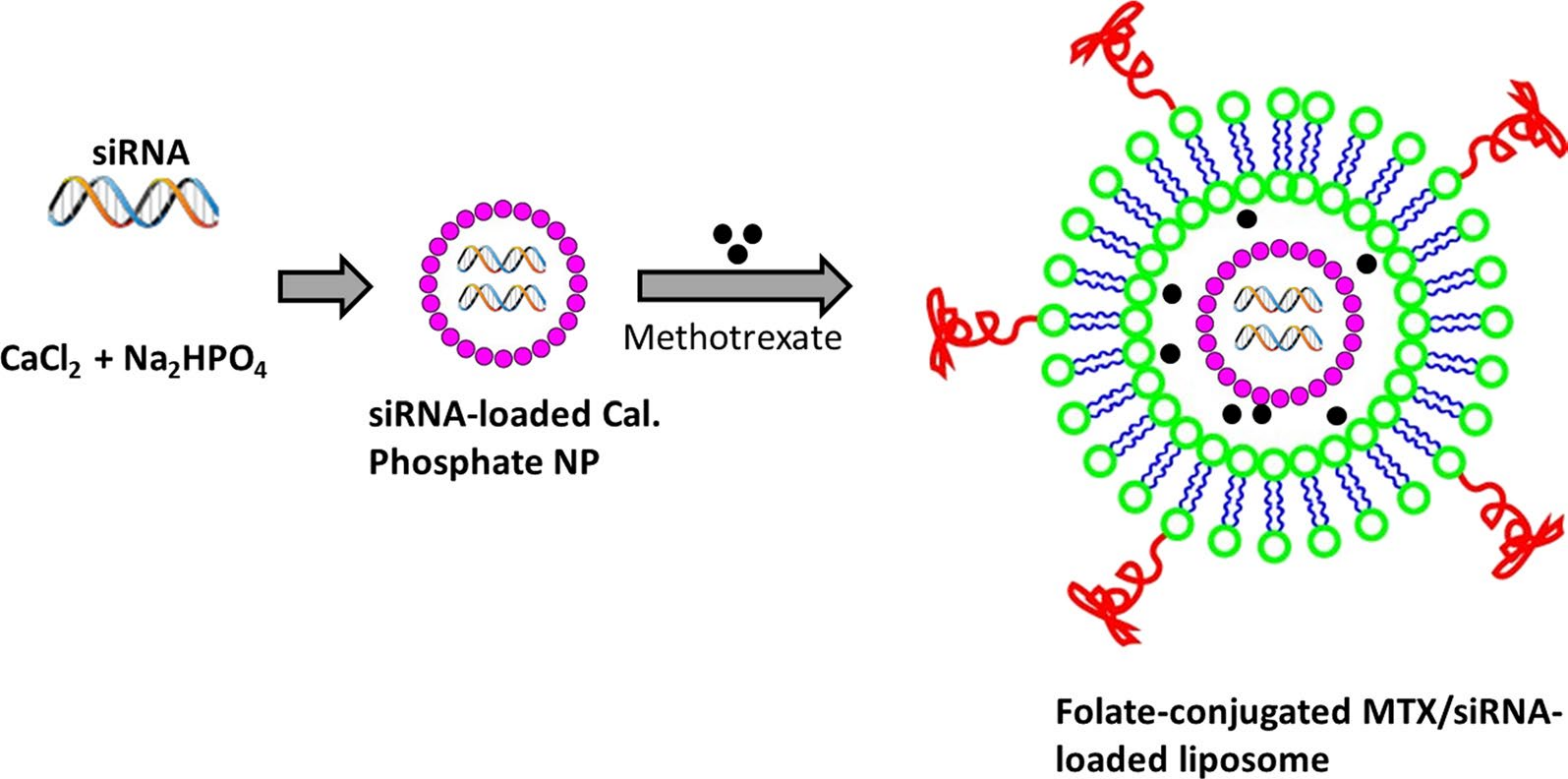
Schematic presentation of preparation of CaP/siRNA NP entrapped methotrexate loaded targeted liposome

NF-kB has been reported to be one of the key therapeutic targets in the management of RA since it is involved in the regulation of inflammatory cytokines such as TNF-α and IL-1β which regulated the RA progression [26]. si-RNA-mediated gene silencing found to be a novel way in the treatment of various inflammatory diseases [27]. In this study, we have selected p65 siRNA that directly targets the NF-kB family member p65 and could potentially inhibit the NF-kB-based inflammatory activities [13]. We expected that siRNA could potentially downregulate the p65 and thereby M1 macrophages re-polarize into M2 macrophages which are involved in tissue repair [13]. In addition, we have combined the MTX along with siRNA to potentiate the RA treatment. The loading of therapeutics in the nanoparticle system is expected to promote the circulation in the blood circulation as well as promote the permeability in the biomembrane [28, 29]. We have prepared the calcium phosphate nanoparticles (CaP) for the delivery of siRNA. We have designed the PEGylated liposome that could load the siRNA/CaP in its core while MTX will be loaded in the lipid shell of the liposomes (Fig. 1).

### Particle size and morphology analysis

The particle size of siRNA-complexed calcium phosphate nanoparticles (siRNA/CaP) and F-siRML was evaluated by dynamic light scattering (DLS) technique. The siRNA/CaP showed an average particle size of around ~ 45 nm and whereas F-siRML showed an average size of ~ 170 nm (Additional file 1: Figure S1a). A small particle size of siRNA/CaP was expected as they are nanocomplexes formed due to the electrostatic interactions. The surface charge after the complex formation was 3.8 ± 0.39 mV while the final surface charge after the lipid coating stood at − 23.6 mV. The increase in particle size after liposome encapsulation was due to the assembly of lipid bilayer around the complex and folate conjugation on the surface. In all case, a particle size of less than 200 nm would be beneficial to circulate in the systemic compartment that might accumulate gradually in the diseased site in a time-based manner [30]. A small particle size of siRNA/CaP (< 100 nm) complex was formed during the electrostatic interactions between siRNA and CaP. A nanosized siRNA/CaP complex will allow the easy encapsulation in the liposome. The particle shape was further confirmed by transmission electron microscopy (TEM) (Additional file 1: Figure S1b). As seen, siRNA/CaP were nanosized and present as a spherical outfit in the copper grid. F-siRML showed typical spherical shaped particles and distinctly separated from each other. A stable spherical particle might be attributed to the presence of PEG on the surface that might maintain the steric balance. The stability analysis of F-siRML was analyzed in PBS and FBS media. As shown, F-siRML showed excellent stability throughout the study period. The particle size of F-siRML insignificantly changed for the study period indicating its excellent stability and structural integrity.

### Gel electrophoresis

To investigate interactions of CaP with siRNA, agarose gel electrophoresis studies were carried out at different N/P ratios (Additional file 1: Figure S2). Results revealed that siRNA was firmly combined with CaP when the N/P ratio was more than 3 and complete complexation was observed at N/P ratio of 5.

### In vitro drug release

**Fig. 2:**
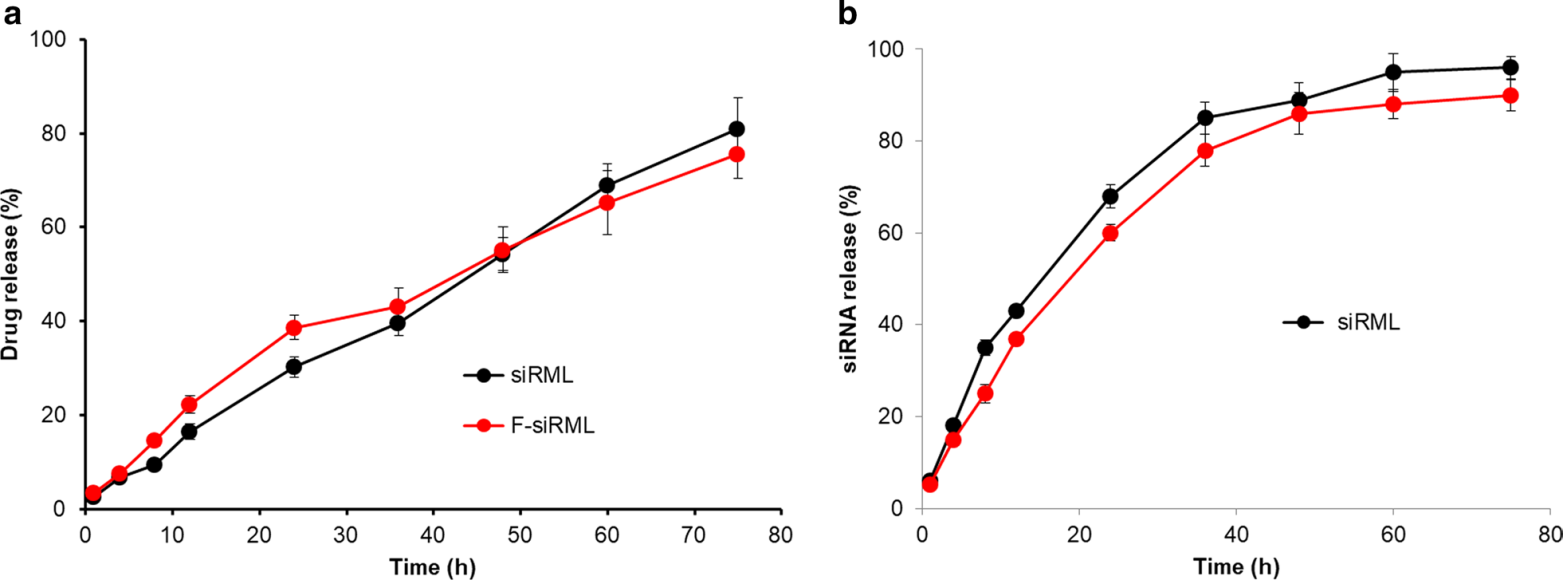
**a** In vitro drug release of methotrexate from siRML and F-siRML. The drug release study was performed in PBS (pH 7.4) and the amount of drug release was estimated by HPLC analysis; **b** in vitro drug release of siRNA from both the formations

The in vitro drug release study was performed in pH 7.4 in order to simulate the intracellular pH conditions (Fig. 2a). As shown, continuous release of drug was observed from both the nanoparticle system. A steady drug release pattern was observed with around 40% of drug released within 24 h. The release of drug continued until the end of the study period. Approximately, 80% of drug released at the end of 75 h indicating a sustained release nature of the delivery system. The release of MTX from both the formulations showed a sustained drug release pattern. At no point during the release study, we observed the burst release pattern indicating the drug was stably loaded in the lipid matrix of the nanoparticle system. During the preparation of MTX-loaded nanoparticle complex, it is believed that most of the drug is interspersed in the lipid matrix rather than on the surface. Therefore, no burst release of drug from NP. It is worth noting that no statistical difference was observed from siRML and F-siRML indicating that a thin-layer of folic acid did not inhibit the release of drugs from the inner nanoparticles. A slow release of drug in the RA site might benefit the continuous exposure of therapeutics and thereby therapeutic efficacy will increase.

We have performed the additional experiment on siRNA release from the formulations (Fig. 2b). For this purpose, FAM-siRNA was loaded and release was observed for 75 h. Contrary to drug release, more than 60% of siRNA was released within 24 h study period. The faster release of siRNA could targets the NF-kB family member p65 and could potentially inhibit the NF-kB-based inflammatory activities. No significant difference in siRNA release was observed between siRML and F-siRML.

### Cellular uptake in inflammatory conditions

**Fig. 3:**
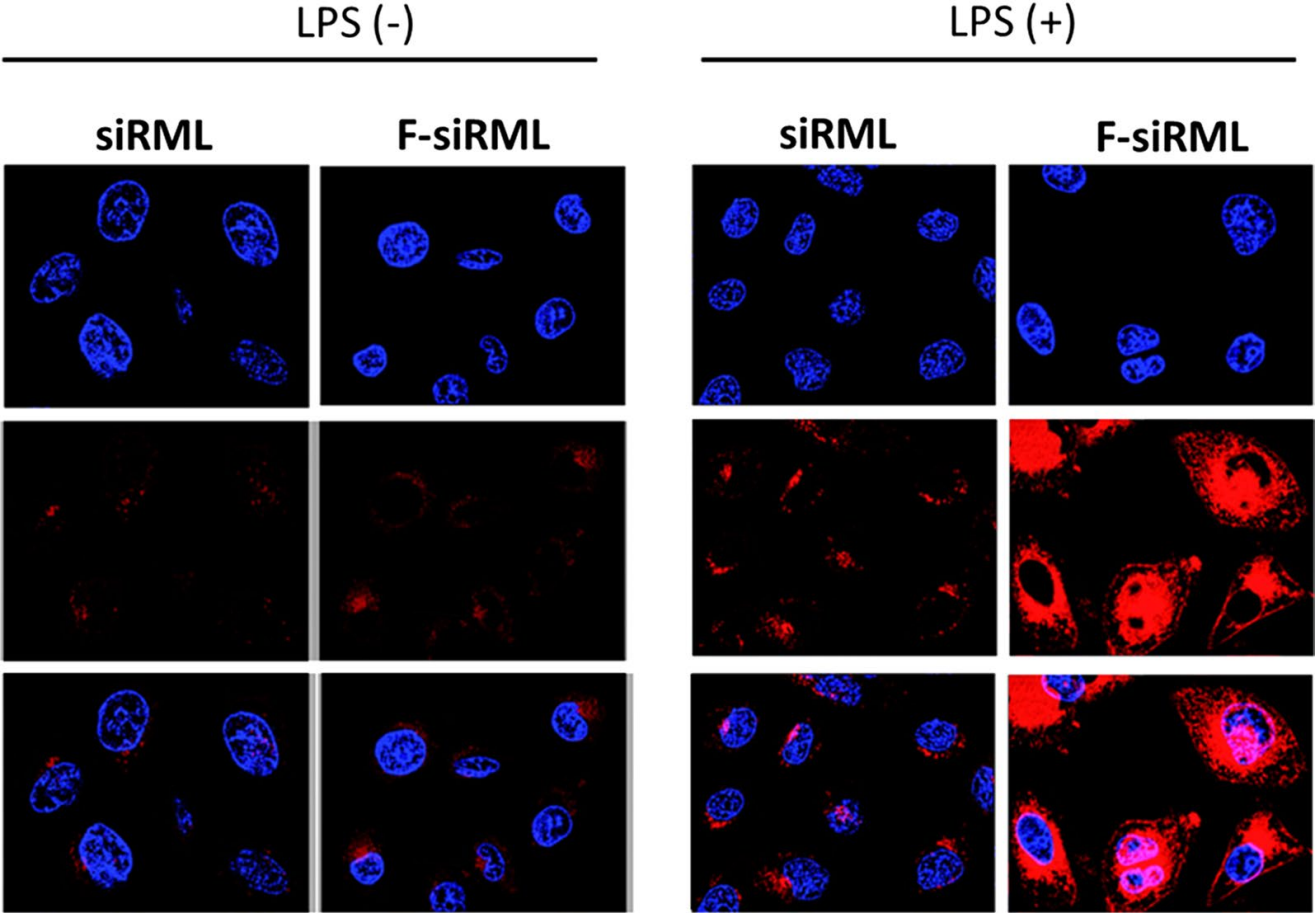
Cellular uptake of siRML and F-siRML in RAW 264.7 macrophage cells in the presence and absence of lipopolysaccharide. The nucleus was stained with DAPI and the liposome was loaded with rhodamine B. The uptake of formulation by RAW 264.7 macrophage cells after activation by LPS (LPS +) or not treated with LPS (LPS −)

The nanoparticle internalization in the macrophages in inflammatory and non-inflammatory conditions was tested in Raw 264.7 cells (Fig. 3). For this purpose, macrophage cell was treated with lipopolysaccharide (LPS) in order to simulate the inflammatory conditions while another macrophage group was untreated with LPS. As the macrophage cell overexpress folate receptors, relative cellular uptake of siRML and F-siRML was tested. Two important observations were noted; first, enhanced fluorescence was observed in LPS-activated cells compared to that of non-LPS activated cells. As clearly shown, substantial accumulation of F-siRML was observed in LPS-activated macrophages. A strong red fluorescence at a λ_ex_ 553 nm and λ_em_ 627 originated from the rhodamine B loaded in the nanoparticles while the nucleus was stained with DAPI as a nuclear-staining dye. Second, presence of targeting ligand (folate) enhanced the cellular uptake compared to non-targeted nanoparticles. The result was in the expected line that folate receptor overexpressed macrophages will preferentially internalize the nanoparticle conjugated with folic acid ligand [31]. The activated macrophages are generally present in the RA and osteoarthritis and cancers and folate-targeted nanoparticle enables the effective accumulation of therapeutics in the diseased site [32]. Overall, higher internalization in LPS-activated macrophages was attributed to non-opsonic phagocytosis of F-siRML and active targeting of folate receptors.

We have carried out an in vitro study on the cell viability of RAW 264.7 cells (Additional file 1: Figure S3). RAW 264.7 cells were treated with various concentrations of blank nanoparticles and incubated for 24 h. As seen, the blank nanoparticles did not exhibit any significant killing effect on the macrophage cells even at higher concentrations indicating the inertness of the prepared nanoparticles.

### Therapeutic efficacy of F-siRML in vivo

**Fig. 4:**
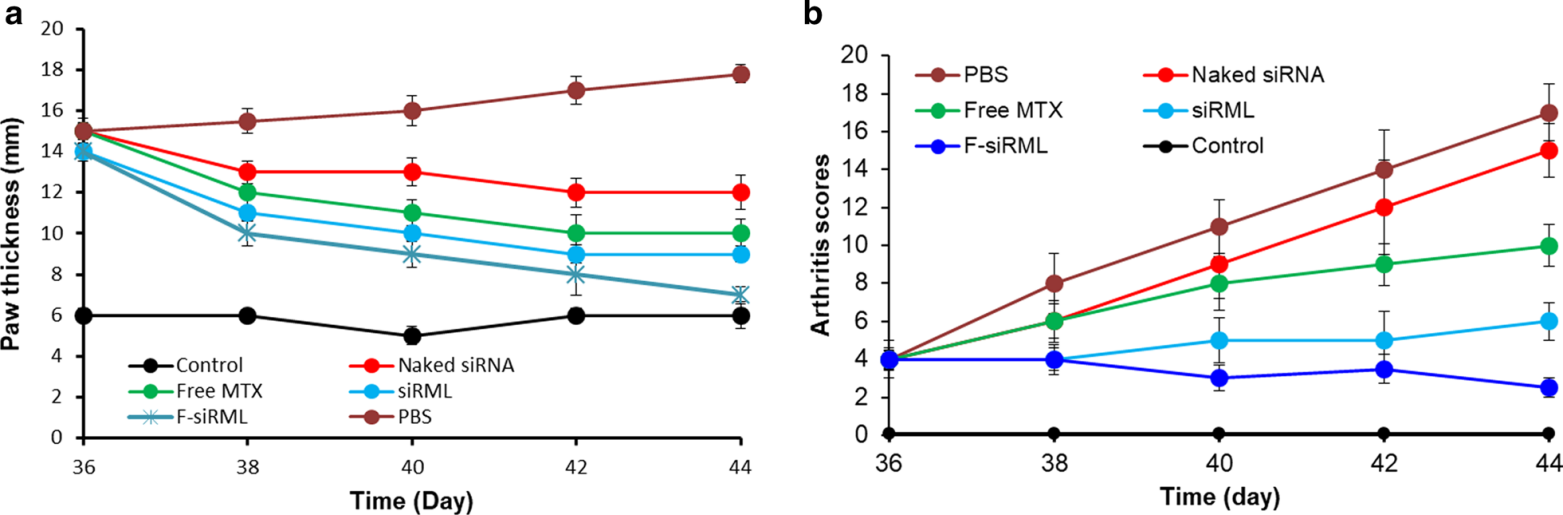
The therapeutic efficacy of siRML and F-siRML was evaluated in arthritic mice. **a** Hind paw thickness, **b** arthritic scores. The results are presented as mean ± SD

The therapeutic efficacy of different formulations was evaluated in arthritic mouse. After development of arthritis sign in the mice, various formulations including naked siRNA, MTX, siRML and F-siRML were administered via intravenous injection. The therapeutic efficacy of formulations was assessed in terms of paw thickness and arthritic scores (Fig. 4). As seen, no antirheumatic effect was observed for naked siRNA and no significant difference was observed between PBS and siRNA treated group indicating that gene by itself will be ineffective. MTX treatment does reduce the paw thickness however it showed relatively less effectiveness than the formulations. As expected, F-siRML significantly suppressed the paw thickness suggesting its excellent therapeutic efficacy. Furthermore, F-siRML (p < 0.001) was significantly more effective compared to that of siRML treated animal group. Additionally, arthritis score was measured and observed that PBS and siRNA treated group showed constant increase in the arthritis score as the disease progressed, while progression was slower in MTX treated group. Consistent with the paw thickness, F-siRML showed no increase in arthritis score and maintained the same joint score throughout the study period. The excellent therapeutic efficacy of F-siRML was mainly attributed to the synergistic combination of siRNA + MTX that might act by different biological pathways. In addition, nanoparticulate encapsulation of therapeutics might increase its accumulation in the RA and presence of folate targeting ligand further increased the cellular permeability and thereby therapeutic efficacy [10]. Recently, it was demonstrated that inflamed tissues such as in RA has enhanced vascular permeability that allows the small particles to extravasate via enhanced permeation and retention effect (EPR) [33]. After internalization via EPR effect, particles are taken up by actives cells such as dendritic cells and macrophages in the synovium [33]. Therefore, a nanosized particle such as in our case could easily extravasate the inflamed cells in RA.

**Fig. 5:**
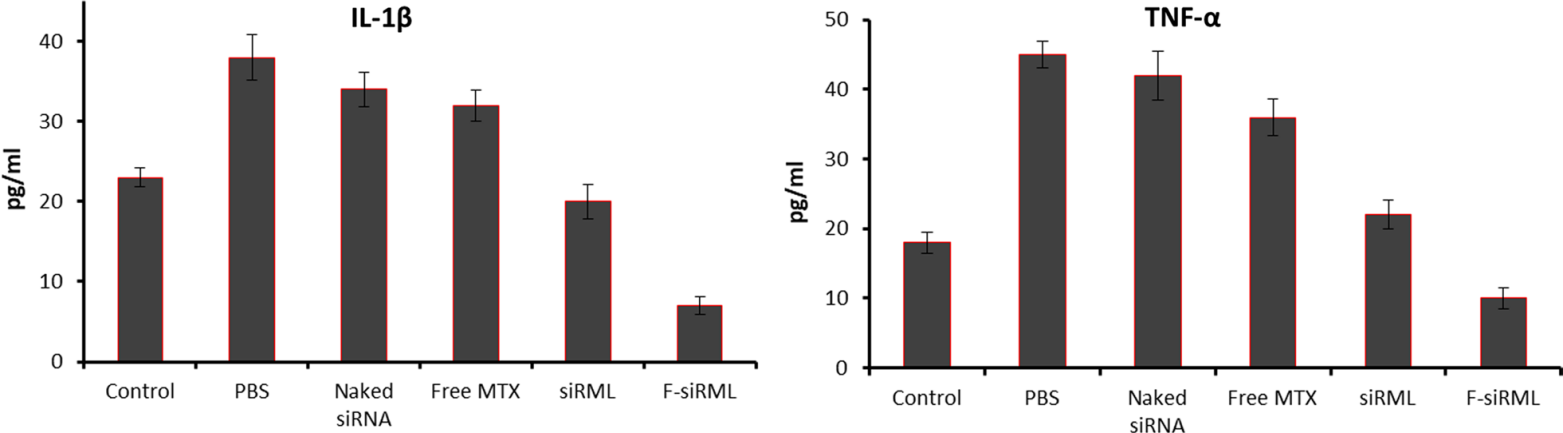
Analysis of levels of pro-inflammatory cytokines from the serum. The arthritic mice were injected withal formulations and serum was collected from mice at the specified interval (day 44) after the administration

In addition, serum levels of TNF-α and IL-1β were measured which are important markers or signs for the development of RA [34] (Fig. 5). As seen, PBS and siRNA treated groups showed elevated levels of TNF-α and IL-1β indicating the disease progression in RA. As expected, siRML and F-siRML significantly decreased the levels of TNF-α and IL-1β compared to that of either PBS or siRNA or MTX indicating the potential of combination therapeutics as well as drug delivery system such as CaP/liposome carrier [11].

### Safety of siRML and F-siRML in arthritic mice

**Fig. 6:**
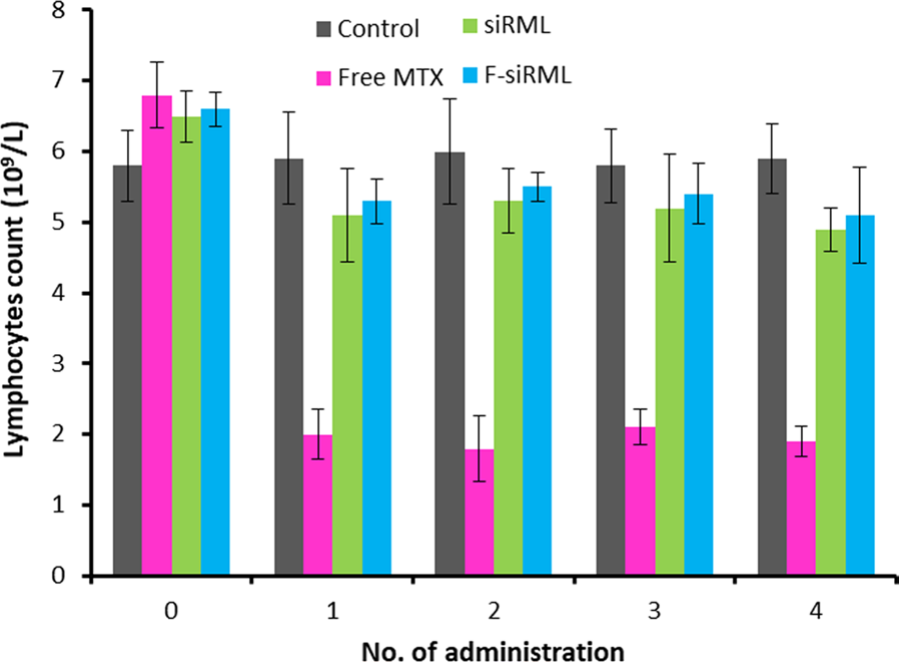
Adverse events of free drug and siRML and F-siRML were evaluated in terms of lymphocyte count. The blood was collected after every administration of the formulations. The blood was collected 5 h after the administration of the formulations

In this study, we have evaluated the levels of lymphocyte as an ideal parameter for the adverse effect of the formulations (Fig. 6). As shown, free MTX drastically reduced the lymphocyte count compared to control indicating its toxicity concern in the body. As expected, siRML and F-siRML did not show any decrease in the lymphocyte count indicating that drug encapsulation in the liposome prevented its release in the systemic circulation and thereby avoided the adverse effect of MTX [35]. Therefore, siRML and F-siRML provides unique benefits of excellent therapeutic efficacy with excellent safety profile in the arthritic mice.

NF-kB system is the important therapeutic target for inflammatory response and therefore it is one of the most studied systems [26]. Once NF-kB system is activated by means of stimulus signal then it will pave way for the release of various inflammatory cytokines such as TNF-α and IL-1β. So far, many small molecules have targeted to control the secretion of aforementioned cytokines however most of the therapeutics resulted in severe adverse effects. In addition, blockage of inflammatory cytokines alone will not prevent the activation of NF-kB system [36]. Therefore, a combination system is warranted which acts by two different mechanisms [12]. Overall, results clearly show that combination of siRNA + MTX might increase the therapeutic efficacy in RA. It can be expected that siRNA targeting p65 might suppress its translocation and thereby prevent the release of pro-inflammatory cytokines from macrophages. In vivo results are evident that combination of siRNA + MTX in nanocarrier could slow down the progression of RA without affecting the physiological parameters in the body.

## Conclusion

Overall, we have successfully demonstrated that the combination of siRNA and methotrexate in a calcium phosphate/liposome-based hybrid nanocarrier could effectively treat the RA. We have showed that folate receptor-targeted nanocarrier system significantly suppression the arthritis progression in mice model. Substantial accumulation of F-siRML was observed in LPS-activated macrophages. These kind of activated macrophages are generally present in the RA and osteoarthritis and folate-targeted nanoparticle enables the effective accumulation of therapeutics in the diseased site. The combinational nanoparticles effectively blocked the NF-kB signaling pathways and reduced the expression of pro-inflammatory cytokines. Furthermore, siRML and F-siRML did not show any decrease in the lymphocyte count indicating that it can avoid the adverse effect of MTX. Therefore, siRML and F-siRML provides unique benefits of excellent therapeutic efficacy with excellent safety profile in the arthritic mice and could be a promising approach in the treatment of rheumatoid arthritis.

## Materials and methods

1,2-Distearoyl-sn-glycero-3-phosphatidylcholine (DSPC), 1,2-distearoyl-sn-glycero-3-phosphoethanolamine-*N*-[meth-oxy(polyethyleneglycol)-2000] (DSPEPEG2000), 1,2-distearoyl-sn-glycero-3-phosphoethanolamine-*N*-[meth-oxy (polyethyleneglycol)-2000-folate (DSPE-PEG2000-folate)], and cholesterol were purchased from Avanti Polar Lipids, China. Pluronic F68, calcium chloride, and sodium phosphate were purchased from Sigma-Aldrich. A siRNA duplex was designed by Guangzhou RiboBio Co., Ltd. (Guangzhou, People’s Republic of China). It is a 21 bp double-stranded RNA oligos with dTdT 3′ overhangs and has sequences as follows: (sense) 5′-GGACAUAUGAGACCUUCAAdTdT-3′; and (antisense) 5′ UUGAAGGUCUCAUAUGUCCdTdT-3′.

### Preparation of siRNA/calcium phosphate complex

The siRNA/CaP was prepared by mixing 75 mM of CaCl_2_, Pluronic F68 (1%) and siRNA (100 µg/ml) and incubated for 15 min. To this solution, mixture of NaHPO_4_ (6 mM), Pluronic F68 (1%) and sod. citrate (24 mM) was added and stirred for 1 h. The conjugation efficiency of siRNA to the nanoparticle was determined by gel retardation assay in 2% agarose gel containing ethidium bromide dye and run in a tris–acetate running buffer at 80 V for 20 min. Free siRNA was used as a loading control. The siRNA retardation was photographed using INFINITY 3026 gel image machine (Vilber Lourmet Deutschland GmbH, Germany).

siRNA/CaP-loaded folate conjugated liposome was prepared by thin-film hydration technique. Briefly, DSPC, cholesterol, DSPE-PEG, and DSPE-PEG-Fol (4:1.2:0.15:0.04 molar ratios) and methotrexate (20% of total lipid) were added in chloroform and the organic solvent was removed by rotary evaporator (RE-52C, Shanghai Yaguang Instrument Co, Ltd). The resulting lipid film was hydrated with siRNA/CaP solution wherein siRNA/CaP complex loaded in the inner core while it is surrounded by lipid film. The liposome was extruded 21× using a mini-extruder at room temperature. The so-formed liposome was dialyzed and stored until further use. To separate the unencapsulated MTX, liposome was passed through Sephadex G-50 column and PBS was used. The free MTX separated from column was analyzed using an HPLC. Cecil CE1100 liquid chromatography system equipped with a CE 1000 pump, a CE1200 variable wavelength UV–Visible detector and a Rheodyne 20 µl loop injector system was used (Cecil Instruments, England). The chromatography column was a 250 × 4.6 (i.d)-mm Spherisorb ODS2 with 5-μm particles (Hichrom, England). The mobile phase, consisting of 50 mM sodium acetate buffer (pH 3.6)–acetonitrile, 89/11 (v/v) was used at a flow rate of 1 ml/min.

### Particle size and morphology analysis

The particle size of liposome was determined by photon correlation spectroscopy using Malvern Instruments, UK). The liposome formulation was suitable diluted with distilled water and experiments were performed at room temperature (24 °C). The experiments were performed in triplicate with 15 runs in each measurement. The morphology of the particle was analyzed by transmission electron microscope (TEM; JEM1200, Japan). The particles were stained with 2% phosphotungistic acid as a negative staining and the particles are dried. The carbon-coated copper grid was then viewed under the electron microscope.

### In vitro drug release

The in vitro drug release of F-siRML was analyzed in PBS (pH 7.4). The 1 ml of liposome formulation was packed in a dialysis membrane (MW ~ 3500 Da) and borders were sealed and placed in a large volume of respective buffer containing 1% Tween 80. The study was performed at 37 °C in 100 rpm. At predetermined time interval, samples are withdrawn and replaced with equal volumes of fresh buffer. The amount of drug released at each time interval was determined by HPLC analysis as mentioned above.

The release of FAM-siRNA from siRML and F-siRML was studied in PBS. The formulations were loaded in dialysis membrane (MW ~ 25,000 Da) and sealed and placed in PBS (1% Tween 80) at 37 °C. At given times, the tubes were centrifuged (18,000*g* for 15 min); the supernatants were collected for analysis. The amount of siRNA in the supernatant was determined using a calibration curve and the release was calculated as (1 − (FAM-siRNA_supernatant_/FAM-siRNA_encapsulated_)) 100%. The concentration of released Cy3-siRNA was measured using the FLUOstar OPTIMA plate reader.

### Targeting efficiency of F-siRML in RAW 264.7 cells

The RAW 264.7 cells were seeded in a 6-well plate at a density of 3 × 10^5^ per each well of the plate and incubated for 24 h. Next day, cells were incubated with siRML and F-siRML and incubated for 3 h at 37 °C in incubator. The cells were then washed stained with DAPI as a nuclear staining dye. The liposome was loaded with rhodamine B as a tracking agent. After the staining was removed and washed, cells were fixed with 4% paraformaldehyde for 10 min. The cells were then viewed with confocal laser scanning microscope (CLSM).

### Collagen-induced arthritis mouse model

All animal experiments were approved by the Institutional Animal Health Care Committee of Luoyang Orthopedic Hospital of Henan Province. The DBA/1 J mice aged 6-weeks was intradermally injected with a mixture (100 µl) of 100 µg of bovine CII (2 mg/ml) and equal volume of Freund’s Adjuvant (5 mg/ml). After 21 days, a booster injection containing a mixture of 100 µg of bovine CII (2 mg/ml) and equal volume of Freund’s Adjuvant was injected at the base of tail. Special care was taken for the mice during the course of arthritis development.

### Arthritic scores and paw thickness in arthritic mice

After 5 weeks of arthritis development, mice with arthritis were randomly divided into 5 groups with 6 mice in each group. On days 36, 38, 40, 42 and 44, arthritis mice were intravenously injected with naked siRNA, MTX, siRML and F-siRML, respectively. A dose 0.6 mg/kg MTX and a dose of 0.4 mg/kg of siRNA were selected for the administration to diseased mice. The untreated mice were considered as a control to compare the treatment groups. The severity of arthritis was measured in each limb of mice using the following scoring system: 0—no evidence of erythema or swelling; 1—mild swelling or erythema which are largely present in joints; 2—mild swelling or erythema that extends from ankle to tarsals; 3—erythema and moderate swelling that extends from the ankle to metatarsal joints; 4—erythema and severe swelling encompassing the ankle, foot and digits, or ankyloses of the limb. The scores of animals were measured on alternative days. The paw thickness was also measured at the same time using an Vernier caliper.

On day 44, blood was collected surgically form each group and the levels of pro-inflammatory cytokines including TNF-α and IL-1β were measured using ELISA test as per the manufacturer’s guidelines (ebioscience, San Diego, CA, USA).

### Adverse effects in arthritic rats

On days 36, 38, 40, 42 and 44 after the administration of formulations, arthritis mice were monitored for possible adverse effects. For this purpose, blood was collected and lymphocyte count was performed as per the established protocols. Precisely, bloods were collected before the first administration of various formulations and then the blood was repeatedly collected after every administration of formulations. The blood was collected approximately 5 h after the administration of the formulations.

### Statistical analysis

The results are presented as mean ± SD and performed in triplicate unless and otherwise reported specifically for individual experiments. Differences between two groups were performed by Student’s *t* test and multiple groups were performed by one-way analysis of variance (ANOVA). A minimum difference of p < 0.05 was considered statistically significant.

## Additional file

**Additional file 1: Figure S1.** (A) Particle size analysis of CaP/siRNA NP and F-siRML. The particle size were measured by dynamic light scattering analysis; (B) morphology analysis of CaP/siRNA NP and F-siRML by transmission electron microscope (TEM). **Figure S2.** Gel retardation assay of CaP/siRNA NP at different N/P ratio. Naked siRNA was taken as a control. **Figure S3.** In vitro cell viability of blank nanoparticles in RAW 264.7 cells. The cell viability assay was performed by MTT assay protocol.

## Abbreviations

MTX: methotrexate
RA: rheumatoid arthritis
FA: folic acid
siRNA: small-interfering RNA
CaP: calcium phospate
siRML: siRNA and MTX-loaded liposome
F-siRML: folate/siRNA and MTX-loaded liposome
