# Supplementary material for: Combination of NF-kB targeted siRNA and methotrexate in a hybrid nanocarrier towards the effective treatment in rheumatoid arthritis
Source: J Nanobiotechnology. 2018 Jul 30;16:58. doi: 10.1186/s12951-018-0382-x (PMC6065064; doi:10.1186/s12951-018-0382-x)
Supplement: Supplementary file 1 — Additional file 1: Figure S1. (A) Particle size analysis of CaP/siRNA NP and F-siRML. The particle size were measured by dynamic light scattering analysis; (B) morphology analysis of CaP/siRNA NP and F-siRML by transmission electron microscope (TEM). Figure S2. Gel retardation assay of CaP/siRNA NP at different N/P ratio. Naked siRNA was taken as a control. Figure S3. In vitro cell viability of blank nanoparticles in RAW 264.7 cells. The cell viability assay was performed by MTT assay protocol. [file 12951_2018_382_MOESM1_ESM.docx]

**Combination of NF-Kb targeted siRNA and methotrexate in a hybrid nanocarrier towards the effective treatment in Rheumatoid arthritis**

**Running Header:** NF-Kb-siRNA and methotrexate in rheumatoid arthritis

Weifeng Duan^1^*, Huan Li^2^

^1^Department of Limb Function Rehabilitation, Luoyang Orthopedic Hospital of Henan Province, Luoyang, Henan Sheng, China, 471000

^2^Nursing College, Henan University of Chinese Medicine, Zhengzhou, Henan Sheng, China, 450000

Weifeng Duan - [DarrellxTurnerqh@yahoo.com](mailto:DarrellxTurnerqh@yahoo.com)

Huan Li - ha3bjxt@163.com

*Corresponding author:

Weifeng Duan, Department of Limb Function Rehabilitation， Luoyang Orthopedic Hospital of Henan Province, Luoyang, Henan Sheng, China, 471000

Tel/Fax: 0371-85965123

Email: [DarrellxTurnerqh@yahoo.com](mailto:DarrellxTurnerqh@yahoo.com)


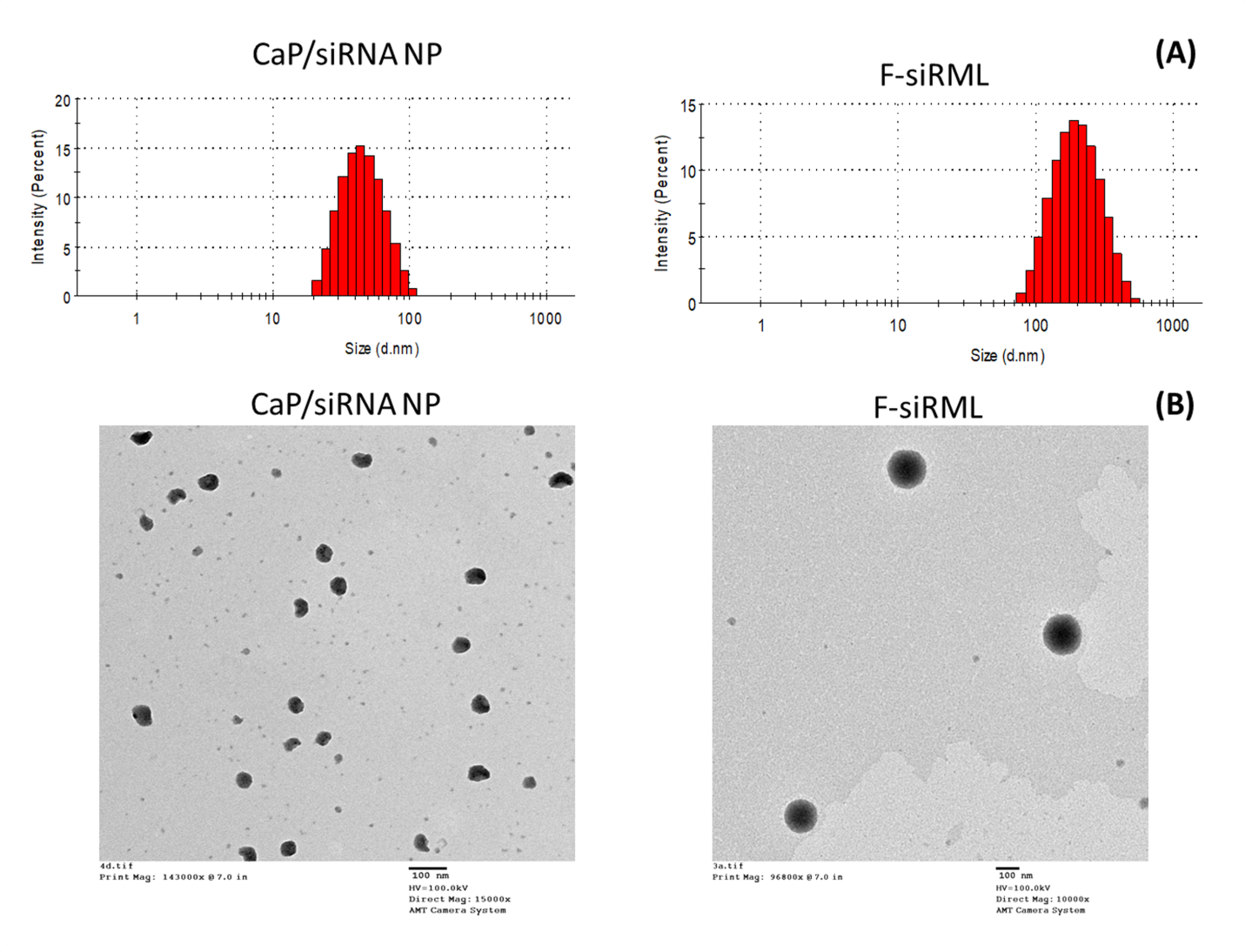


Figure S1: (A) Particle size analysis of CaP/siRNA NP and F-siRML. The particle size were measured by dynamic light scattering analysis; (B) morphology analysis of CaP/siRNA NP and F-siRML by transmission electron microscope (TEM)


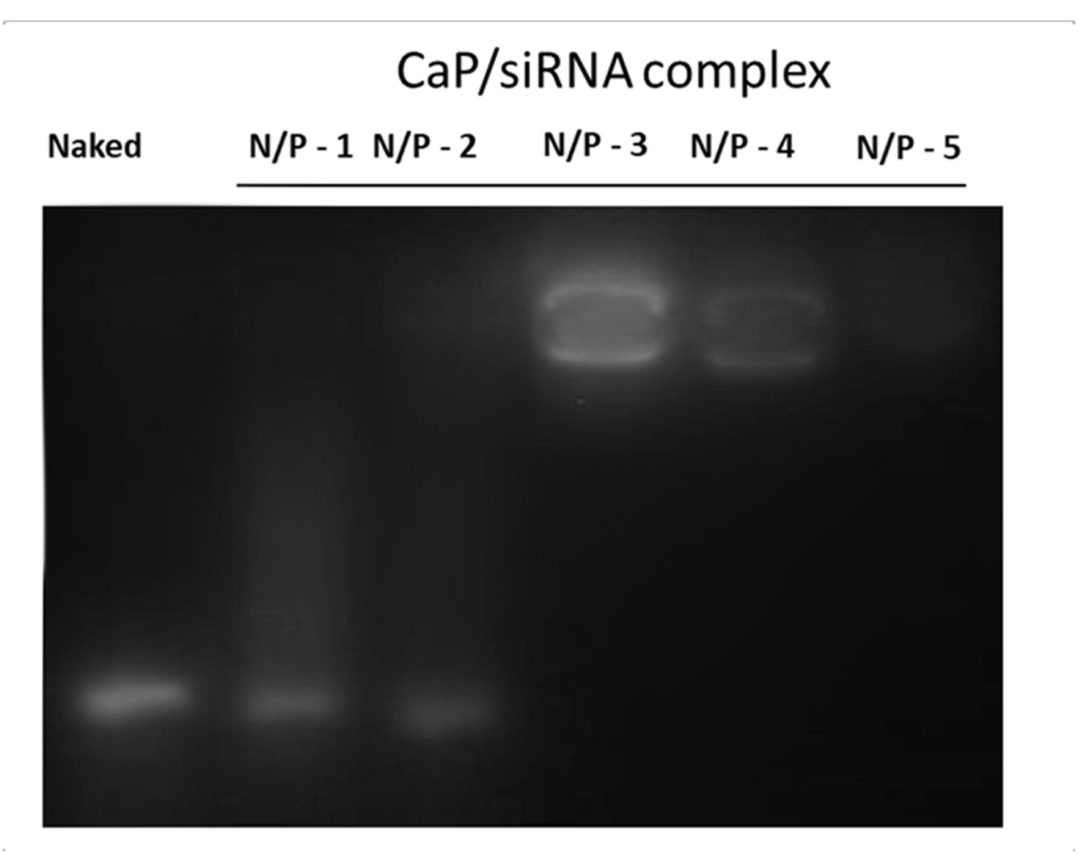


Figure S2: Gel retardation assay of CaP/siRNA NP at different N/P ratio. Naked siRNA was taken as a control

Figure S3: In vitro cell viability of blank nanoparticles in RAW 264.7 cells. The cell viability assay was performed by MTT assay protocol.
